# Sphaeropsidin A covalently binds to Cys 151 of Keap1 to attenuate LPS-induced acute pneumonia in mice

**DOI:** 10.1016/j.redox.2025.103621

**Published:** 2025-03-27

**Authors:** Kang Yang, Qing-Tong Han, Rong-Xue Xing, Zhi-Ying Li, Lin-Tao Xu, Lu-Zhou Chen, Lan Xiang, Dong-Mei Ren, Qing-Wen Hu, Xiao-Ning Wang, Tao Shen

**Affiliations:** a State Key Laboratory of Discovery and Utilization of Functional Components in Traditional Chinese Medicine, Shandong University, Jinan, 250012, China; b Key Lab of Chemical Biology (MOE), School of Pharmaceutical Sciences, Shandong University, Jinan, 250012, China; c Shandong Engineering Research Center for Traditional Chinese Medicine Standard, School of Pharmaceutical Sciences, Shandong University, Jinan, 250012, China; d Shandong Key Laboratory of Bioactive Components and Translational Research of Traditional Chinese Medicine, Jinan, 250012, China

**Keywords:** Acute pneumonia, Sphaeropsidin A, Keap1, Nrf2, NF-κB, NLRP3 inflammasome

## Abstract

**Introduction:**

Kelch ECH-associating protein 1 (Keap1)-Nuclear factor erythroid 2-related factor 2 (Nrf2) axis is crucial for regulating oxidative stress and inflammatory responses in acute pneumonia. Sphaeropsidin A (SA) is a antioxidant diterpenoid isolated from *Sphaeropsis sapinea* f. sp. *cupressi,* discovered as a novel Nrf2 agonist by our research group previously. However, the accurate function and mechanism of SA in treating acute pneumonia are still unknown.

**Methods:**

The therapeutic effect of SA was evaluated in LPS-induced acute pneumonia in mice. The underlying mechanism of action was then analyzed by transcriptomics. The direct target of SA was identified through the synthesis of SA-biotin probe, and the binding amino acid residues were found and verified by LC-MS/MS analysis and site-specific mutation. Finally, knockout mice were employed to verify the mechanism of SA.

**Results:**

Our data indicated that SA significantly inhibited LPS-induced acute pneumonia in mice via up-regulating Nrf2, inhibiting NLRP3 inflammasome and NF-κB activation, and identified Keap1 as the direct target of SA. Specifically, the effective dose of SA in mice was only 2 mg/kg. SA selectively covalent bound to Keap1 in cysteine 151 residue (Cys151). SA mediated the activation of Nrf2 and reduced the level of ROS, thereby inhibiting the NF-κB and NLRP3 inflammasome. Besides, SA formed hydrogen bond with ASP48 of ASC, blocking its oligomerization and inhibiting the activation of NLRP3 inflammasome.

**Conclusion:**

This study indicates that SA might be a new covalent molecule of Keap1 to activate Nrf2, and is a promising drug candidate or lead molecule for the therapy of acute pneumonia through regulating Nrf2/NF-κB/NLRP3 inflammasome axis.

## 1 Introduction

Pneumonia, as a common acute respiratory infectious disease, has already become the sixth-leading cause of death overall worldwide [1]. In general, the pathological process of pneumonia was driven by the inflammatory response and oxidative stress, which were induced by foreign pathogens and pollutants such as viruses, bacteria, cigarette smoke, and so on [2,3]. Accumulating evidence underscored that, the generation of reactive oxygen species (ROS) played a role in activating downstream inflammatory pathways, involving accelerating the release of inflammatory factors [4,5]. Especially, COVID-19 patients exhibited marked abnormalities in oxidative stress levels and inflammation indicators [6,7]. Consequently, targeting oxidative stress and inflammatory response emerges as a potentially effective strategy for both preventing and treating pneumonia.

Nuclear factor erythroid 2-related factor 2 (Nrf2), a key transcription factor, was involved in the regulation of redox homeostasis, including ROS [8]. Besides, NF-κB (Nuclear transcription factor-κB) and NLRP3 (NOD-like receptor 3) inflammasome also played crucial roles in cellular inflammation responses [9,10]. Studies found that the up-regulation of Nrf2 reduced the pro-inflammatory response, regulated by NF-κB transcription [11]. Meanwhile, Nrf2 negatively regulated the activation of NLRP3 inflammasome during the regulation of ROS [12,13]. Given the strong corelation among Nrf2, NF-κB, and NLRP3, the Nrf2/NF-κB/NLRP3 axis was considered as an efficient therapeutic option for attenuating severe pulmonary inflammation. Beyond that, the interplay between Kelch ECH-associating protein 1 (Keap1) and Nrf2 has assumed considerable importance. Specifically, Keap1 could be assembled with Cullin3 (Cul3) and Rbx1 in the BTB domain into a functional E3 ubiquitin ligase complex (Keap1-Cul3-E3) to regulate Nrf2 [14,15]. When exposed to ROS or electrophilic stress, specific cysteine residues in Keap1, e.g. Cys151, were modified, which would caused conformational changes of Keap1-Cul3-E3 ubiquitin ligase, and Nrf2 ubiquitination was interfered [16,17]. At last, Nrf2 was translocated to the nucleus, and the expression of ARE-mediated downstream genes, e.g. NQO1, GST, GCL, GSH, etc., were further activated [18]. Hence, the discovery of small molecules that could covalently modify cysteine residues in Keap1 is pivotal for harnessing the Nrf2-Keap1 pathway in disease treatment.

Diterpenoids are a group of secondary natural metabolites produced by plants or microorganisms with a wide range of biological activities including anti-inflammatory, antioxidant and antitumor, antibacterial and antiviral effects [19]. Thus, diterpenoids are an important source of discovering drug-lead compounds. Sphaeropsidin A (SA), an isopimarane-type diterpenoid, was firstly found from *Sphaeropsis sapinea* f. sp. *cupressi* in 1996 as a fungal phytotoxin [20]. Only few studies have reported SA's activies as anti-fungal comound against several human fungal pathogens such as *Mucor miehei* and *Rhizomucor pusillus*, as well as an anticancer agent especially against different species of melanomas [21]. However, there weren't any in-depth *in vivo* pharmacological research until now. Our previous research has revealed it as a potent Nrf2 activator with approximately 10-fold potency than that of well-known Nrf2 activator *tert*-butyl hydroquinone (tBHQ), which indicated SA as one of the most active naturally-observed Nrf2 activators [22]. Thus, the pharmacogenetics potential of SA in treating pneumonia and its mechanism are worth studying.

In this study, we comprehensively verified that SA significantly inhibited LPS-induced oxidative stress and pulmonary inflammation in mice. Furthermore, we delved into the mechanisms underlying the effect of SA in inhibiting pneumonia *in vivo and in vitro.* Our research demonstrated that SA bound to Cys 151, the key action site of Keap1, and thereby activating Nrf2. Additionally, we discovered that SA hindered the activation of the NLRP3 inflammasome by blocking ASC oligomerization.

## 2 Materials and methods

### 2.1 Chemicals and reagents

The isopimarane-type diterpenoid SA with a purity >95 % was isolated from the culture medium of *Botrysphaeria laricina* by our laboratory [23]. L-Glutamine was obtained from Solarbio (Beijing, China). Nigericin, 3,4-dihydroxy-benzohydroxamic acid (Didox), and 3-(4,5-dimethylthiazol-2-yl)-2,5-diphenyltetrazolium bromide (MTT) were products of MedChemExpress (NJ, U.S.A.). N,N,N′,N'-Tetramethyl ethylenediamine (TEMED), 4 % paraformaldehyde, Tween-20, Triton X-100, β-mercaptoethanol and glycerol were obtained from Dingguo (Beijing, China). DMEM were purchased from Gibco (CA, U.S.A.). Sulforaphane (SF), tertiary butylhydroquinone (tBHQ), monosodium urate (MSU), adenosine triphosphate (ATP), and lipopolysaccharide (LPS) were obtained from Sigma-Aldrich (MO, U.S.A.).

### 2.2 Cell lines and cell culture

J774A.1 murine macrophages cells, RAW 264.7 murine macrophages cells and normal human bronchial epithelial Beas-2B cells were all obtained from American Type Culture Collection (Manassas, U.S.A.), and Nrf2 knock-down RAW 264.7 murine macrophages cells were a gift from Prof. Tao Zeng (School of Public Health, Shandong University). All cells were cultured in DMEM (Gibco, CA, U.S.A.) complemented with 10 % FBS, 0.29 g/L l-glutamine, 100 U/mL penicillin, and 100 μg/mL streptomycin under a humidified 5 % (v/v) CO_2_ atmosphere at 37 °C.

### 2.3 Cell viability assay

RAW 264.7 cells or J774A.1 cells were seeded in 96-well plates, and exposed to different doses of SA for the indicated time. Then, 20 μL of MTT solution was added to each well, and incubated at 37 °C for 3 h. The supernatant was decanted and dissolved in 100 μL DMSO for 10 min. The cell viability was detected by measuring the absorbance at 570 nm using the Model 680 plate reader.

### 2.4 Luciferase reporter gene assay

Cells were seeded in 24-well plates and transfected with NF-κB-luciferase plasmid and Renilla luciferase plasmid using lipofectamine 2000™ (Invitrogen, CA, USA). The transfected cells were treated with the indicated doses of SA for another 1 h, followed by cotreatment with SA and LPS (1 μg/mL) for an additional 16 h. The firefly and Renilla luciferase activities were measured using the Promega dual luciferase reporter gene assay system (WI, USA).

### 2.5 Immunofluorescence

RAW 264.7 cells were seeded in 35 mm glass bottom dishes with cell coverslips. After exposure to the specified concentration of SA, the cells were fixed for 10 min by acetone/methanol (1:1). Then the cell coverslips were exposed to the primary antibody overnight at 4 °C, and subsequently to Alexa Flour 594 (1:400) for 3 h at R.T. After washing, the cell coverslips were stained with DAPI for 10 min in the darkness. The fluorescence signals were observed by Olympus BX53+DP73 microscope system (Tokyo, Japan).

### 2.6 Measurement of ROS

RAW 264.7 cells were seeded into 35 mm glass bottom and pretreated with the indicated dose of SA for 8 h, followed by cotreatment with LPS (1 μg/mL) for an additional 8 h. The supernatant was removed, and DCFH-DA (10 μM, Keygen Biotech, Nanjing, China) was added and incubated for 15 min. ROS level was detected with the fluorescence intensity at 530 nm after excitation at 488 nm on FACSC alibur flow cytometer (BD biosciences, CA, USA). In addition, the fluorescence signals were photographed with Olympus BX53+DP73 system.

### 2.7 In situ pull-down experiment and target validation

Cells were grown to 70 % confluence in 10-cm dishes and incubated with 10 μM SA-Bio probe at 37 °C with 5 % CO_2_ for 1 h. Cells were harvested, lysed in 0.1 % NP-40/PBS using a probe sonicator, and centrifuged at 15,000 g for 30 min to remove cell debris. The concentrations of the whole proteome were determined by BCA protein assay and normalized to 2 mg/mL. In pull-down experiments, the eluted samples were separated on a 10 % SDS-PAGE gel and immunoblotted with anti-Keap1, Nrf2, and ASC antibody.

### 2.8 Expression and purification of recombinant Keap1 protein

DNA coding sequence of the human Keap1, was subcloned into a pET-28a (+) expression vector. Keap1 protein was expressed using E. coli strain Rosetta (DE3) (Tiangen Biotech (Beijing) Co., Ltd.) in 2 L Luria-Bertani medium induced with isopropyl-β-D-thiogalactoside (1 mM). Protein purification used a Ni-NTA Agarose column. The protein concentration and purity (>85 %) were estimated using NanoDrop™ 2000 spectrophotometers (Thermo Fisher Scientific) and SDS-PAGE (Bio-Rad) respectively. Recombinant Keap1 protein was concentrated and stored at −80 °C.

### 2.9 Binding-site exploration between SA and Keap1

Recombinant Keap1 protein (100 μL, 10 μM) in PBS was incubated with SA (100 μL, 20 μM) for 1 h at room temperature. The resulting mixture was then added 150 μL of 8 M urea and 100 mM DTT, and the mixture was incubated for 1 h at 35 °C. Then the sample was transferred to a 10-kDa cut-off filter (Microcon-10 kDa Centrifugal Filter Unit, Millipore). After centrifugation at 14,000 g for 20 min, the sample was washed with 150 μL of 50 mM NH_4_HCO_3_ at 14,000 g for 20 min. Then 20 mM IAA (150 μL) was added to the filter, followed by further incubation for 1 h in dark. After centrifugation at 14,000 g for 20 min, the sample was washed with 150 μL of 50 mM NH_4_HCO_3_ for three times. Next, 150 μL of 10 mM NH_4_HCO_3_ and trypsin (trypsin: Keap1 protein 1:100, w/w) to the mixture, followed by further incubation at 37 °C for 16 h. The resulting peptides were extracted and desalted for downstream LC-MS/MS analysis.

### 2.10 Nuclear and cytoplasmic extraction

RAW 264.7 cells were seeded into 60 mm glass bottom dishes, treated with indicated doses of SA for 1.5 h, followed by cotreatment with LPS (1 μg/mL) for additional 4 h. Then, the proteins of nuclear and cytoplasmic were separated using nuclear and cytoplasmic protein extraction kit (Bioteke Corporation, Beijing, China).

### 2.11 Real-time reverse transcription-polymerase chain reaction (RT-PCR)

J774A.1 cells or RAW 264.7 cells were seeded into 35 mm glass bottom dishes, and treated by indicated time respectively. The total mRNA was isolated by Trizol reagent (Invitrogen, Carlsbad, CA). Equal amount of total RNA (1 μg) was reverse transcribed into cDNA by PrimeScriptTM RT Reagent Kits with gDNA Eraser (Takara Bio Inc., Japan) and primers on the Mastercycler ep real lex PCR System (Eppendorf, Hamburg, Germany). The specific primer sequences are presented in Supplementary Table 1.

### 2.12 Cellular thermal shift assay (CETSA)

J774A.1 cells were seeded into two 100 mm glass bottom dishes, and treated with SA and DMSO, respectively. The cells were collected and separated into 20 samples, and then heated individually at different temperatures (39-59 °C) for 5 min followed by cooling for 5 min at 37 °C with 3 repetitions. The samples were centrifuged at 15,000×*g* for 15 min at 4 °C, and the supernatant was analyzed by immunoblot analysis.

### 2.13 Plasmids construct and co-immunoprecipitation (co-IP)

The cDNAs for NLRP3 and ASC were obtained using standard PCR techniques from HeLa cells and subsequently inserted into the mammalian expression vector pcDNA3.1(+) (Addgene, Watertown, MA, USA), to introduce the FLAG-tag and Myc-tag respectively. The primer sequences are shown in Supplementary Table 1.

For the co-IP assay, HEK293 T cells were seeded onto 6-well plates and transfected with different plasmids (FLAG-NLRP3 and Myc-ASC) or the same amount of pcDNA 3.1 as control. 42 h after transfection by polyethyleneimine (PEI), SA (2 μM) was added and incubated for another 6 h. Then, cells were lysed on ice with the cold RIPA Lysis Buffer and incubated with a mixture of specific antibodies and beads on a rotary incubator overnight at 4 °C. Following the incubation, the mixture was centrifuged and washed five times with PBS (0.01 % Tween 20), then the samples were collected. Finally, Western blot was performed to display the results with SDS-PAGE, using different antibodies.

### 2.14 Molecular docking analysis

The structural formula of the ligand molecule was downloaded from PubChem data (https://pubchem.ncbi.nlm.nih.gov/), and ligand molecule energy was minimized using Chem3D software. The receptor protein ASC (PDB code: 3J63) was obtained from the PDB database (https://www1.rcsb.org/) and processed using PyMOL software. Vina script was run for molecular binding energy calculation and molecular docking results display. PyMOL software was used to 3D display the ligand-receptor complexes produced by molecular docking to evaluate the reliability of bioinformatics analysis and prediction.

### 2.15 ASC oligomerization assay

J774A.1 cells were seeded into 60 mm glass bottom dishes, stimulated by 300 ng/mL LPS for 3 h, and treated with different doses of SA for 0.5 h. After addition of 3 μM nigericin for 0.5 h and the cells were lysed by NP-40 for 30 min on ice. Lysates were centrifuged at 1000×*g* for 10 min at 4 °C. 2 mM disuccinimidyl suberate (DSS) was added to the resuspended pellets, which were incubated at R.T. for 40 min. Samples were then centrifuged at 10,000×*g* for 10 min at 4 °C. The cross-linked pellets were isolated and analyzed by immunoblot analysis.

### 2.16 In vivo model establishment and drug administration

Male C57BL/6 N mice (age 6-8 weeks, 20-22 g) were purchased from Beijing Vital River Laboratory Animal Technology Co., Ltd. (Beijing, China). The experimental animal protocol (No. 21035) was approved by Laboratory Animal Ethical and Welcare Committee of Shandong University Cheeloo College of Medicine. After 1 week of adaptive feeding, mice were randomly divided into 6 groups: the control group, the LPS exposure group, the tBHQ group (40 mg/kg), the dexamethasone (Dex) group (1 mg/kg), the low-dose group of SA (2 mg/kg), and the high-dose group of SA (4 mg/kg). Nrf2^−/−^ mice and Nrf2^WT^ mice were randomly divided into 5 groups: the control group, the LPS exposure group, the tBHQ group (40 mg/kg), the low-dose group of SA (2 mg/kg), and the high-dose group of SA (4 mg/kg). Dex, tBHQ, and SA were formulated in 5 % EtOH (in normal saline). LPS administered at 10 mg/kg (in normal saline) was aspirated into the lung by a nebulization device (Yuyan Instruments, Shanghai, China). Mice were treated with Dex (1 mg/kg), tBHQ (40 mg/kg) and SA (2 and 4 mg/kg) intraperitoneally for 4 h and then stimulated with LPS (10 mg/kg). Mental status, diet, and fur color changes were observed every day. After LPS exposure for 24 h, mice were sacrificed by cervical dislocation, and lung tissue, blood, and bronchoalveolar lavage fluid (BALF) were collected for subsequent experimental analysis.

### 2.17 Collection and analysis of BALF

The trachea of mice was exposed, and 1 mL of PBS was injected into the trachea with a syringe. Then, the bronchoalveolar lavage fluid was collected and subjected to centrifugal separation at 2000 rpm for 10 min to give supernatant for ELISA detection.

### 2.18 Histopathological evaluation

The right lower lobes of lung tissues were immersed in 4 % paraformaldehyde for 24 h, dehydrated and embedded in paraffin. The paraffin-embedded lung tissues were sliced into 4 μm sections. After deparaffinization, the sections were stained with Masson's trichrome, hematoxylin and eosin (H&E). The images of sections were observed by BX53+DP73 microscope system.

### 2.19 Analysis of lung homogenate

The lung tissues were weighed and homogenized in normal saline (1 g of the tissue in 9 mL of normal saline). Homogenate until lung tissue was completely broken and allowed to stand on ice for 15 min. After centrifugation at 11,000 rpm for 10 min, the supernatant was taken for the measurements. The levels of glutathione (GSH), superoxide dismutase (SOD), malondialdehyde (MDA) amd 8-oxo-7,8-dihydro-2′-deoxyguanosine (8-oxo-dG) were detected by commercial kits.

### 2.20 Statistical analysis

Results were expressed as means ± SD. GraphPad Prism (Version 9.0) was used for statistical analysis. Multiple groups were compared by t-tests, one-way ANOVA analysis or two-way ANOVA. A probability (*p*) value < 0.05 was considered to be statistically significant.

## 3 Results

### 3.1 SA alleviates LPS-induced acute pneumonia in mice

**Fig. 1:**
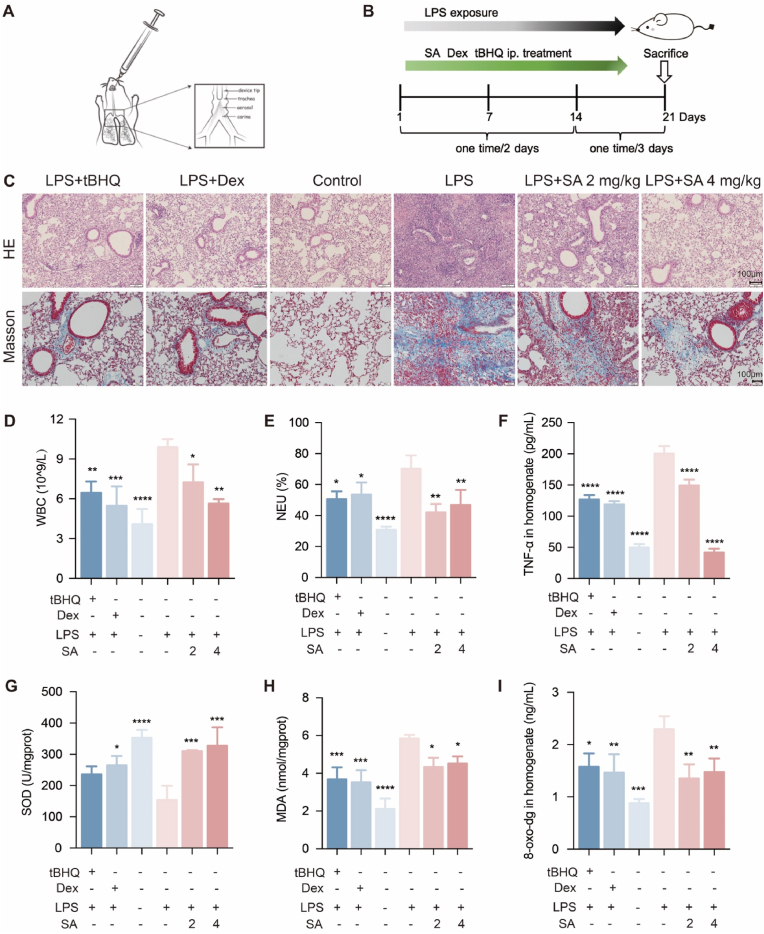
SA alleviated LPS-induced acute pneumonia in mice. (A) Diagram of nebulizer delivery device. (B) Animal test protocol. Dex (1 mg/kg), tBHQ (40 mg/kg), and SA (2 and 4 mg/kg) were injected intraperitoneally for 4 h followed by stimulation with LPS (10 mg/kg) once every two days for 2 weeks, every 3 days for 1 week at the third week. The mice were sacrificed 24 h after LPS nebulization. (C) Lung tissue sections were stained with H&E or Masson. The images of sections were observed under 200× magnification. (D-E) Mouse blood routine. The Mindray automatic hematology analyzer performed a blood routine analysis on collected mouse orbital blood. (F) ELISA kit was used to detect the TNF-α level in BALF. (G-I) ELISA kits were used to detect the levels of SOD, MDA, and 8-oxo-dG in the lung homogenate. Results are expressed as mean ± SD (n = 3), ∗ indicates a significant difference (∗*p* < 0.05, ∗∗*p* < 0.01, ∗∗∗*p* < 0.001).

A lung atomization administration device has been adopted to deliver LPS aerosol into the trachea and lungs of mice, which was used to evaluate the effect of SA against acute pneumonia (Fig. 1A and B). LPS exposure significantly induced pulmanory inflammation, verified by the thickening and collapse of the alveolar wall, and inflammatory cell infiltration [24]. Treatments with SA at 2 mg/kg, as well as the positive controls tBHQ (40 mg/kg) and Dex (1 mg/kg), significantly alleviated LPS-induced pulmanory inflammation and collagen deposition (Fig. 1C). The anti-inflammatory effect of SA was confirmed by the decrease of leukocytes number and the ratio of neutrophils in the blood of mice (Fig. 1D and E). Furthermore, SA relieved the upregulation of TNF-*α* in BALF (Fig. 1F). which also could increase the concentration of SOD and decrease the levels of MDA and 8-oxo-dG, indicating that SA could protect lung tissue from oxidative stress (Fig. 1G–I). Thus, our data demonstrated that SA potently attenuated LPS-induced acute pneumonia *in viv*o, exhibiting excellent antioxidant and anti-inflammatory capacity.

### 3.2 The pathways enrichment and regulation of SA about Nrf2, NF-κB and NLRP3

**Fig. 2:**
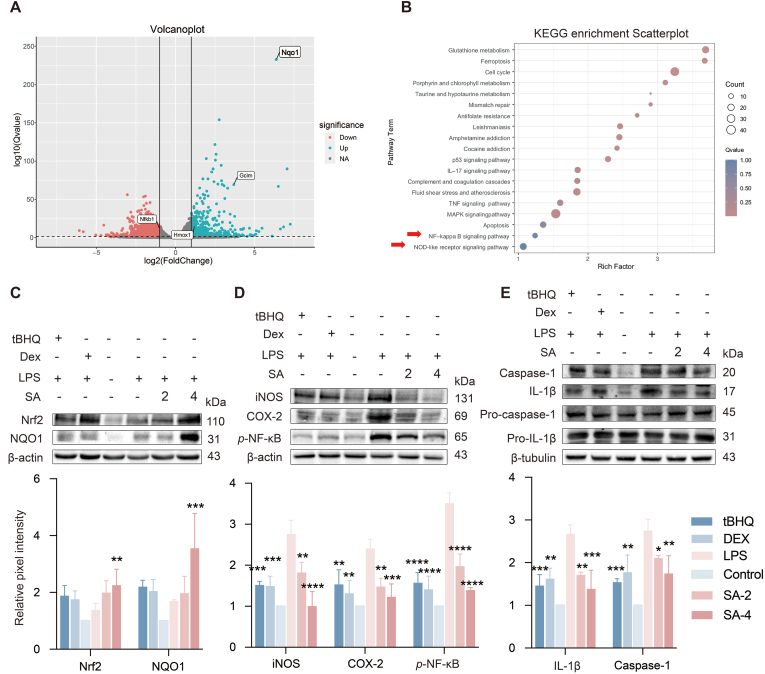
Regulatory pathway of SA enrichment of Nrf2, NF-κB and NLRP3 and validation *in vivo.* (A) Volcano plot showing significantly differentially expressed miRNAs; green pixels represent increased genes, red pixels represent decreased genes, and black pixels indicate genes with no difference. (B) KEGG enrichment scatterplot showed the size and color of each bubble representing the number of genes enriched in the pathway and the significance. (C-E) Effect of SA on Nrf2 and NQO1, iNOS, COX-2 and *p*-NF-κB, Caspase-1 and IL-1β, the protein extraction in the lung tissue was analyzed by western blot. Results are expressed as mean ± SD (n = 3), ∗ indicates significant difference (∗*p* < 0.05, ∗∗*p* < 0.01, ∗∗∗*p* < 0.001).

Based on the anti-inflammatory and antioxidant activity of SA in mice, transcriptome sequencing analysis of J774A.1 cells treated by SA was performed. The relevance to the inflammatory response and oxidative stress was visualized with a volcano plot (Fig. 2A). Compared to the control group, 1419 down-regulated genes and 632 up-regulated genes were observed in the SA-treatment group. Of which, the expressions of the genes NQO1, GCLM, and HMOX1 were regulated by Nrf2, and the gene NF-κB were significantly changed. KEGG enrichment indicated that SA affected the pathways related to the regulation of inflammatory response and oxidative stress, such as glutathione metabolism, ferroptosis, NF-κB and Nod-like receptor signaling pathways (Fig. 2B). These results implied that inhibition of SA against acute pneumonia might be associated with regulations of Nrf2, NF-κB and NLRP3 inflammasome.

The regulations of SA on Nrf2, NLRP3, and NF-κB in the lung were confirmed. Consistent with the results observed in transcriptome sequencing analysis, SA upregulated the protein expressions of Nrf2 and NQO1 (Fig. 2C), reverted LPS-induced upregulation of *p*-NF-κB, iNOS and COX-2 protein expressions (Fig. 2D), and decreased the protein levels of NLRP3-mediated genes, IL-1β and caspase-1 (Fig. 2E). Accordingly, SA inhibited the production of pro-inflammatory factors IL-6 and IL-18 in the BALF (Fig. S1). Taken together, these data suggested that the inhibition of SA on LPS-induced pulmonary inflammation may be related to the regulation of Nrf2, NF-κB and NLRP3 inflammasome.

### 3.3 SA covalently modified Cys151 of Keap1 to activate Nrf2

**Fig. 3:**
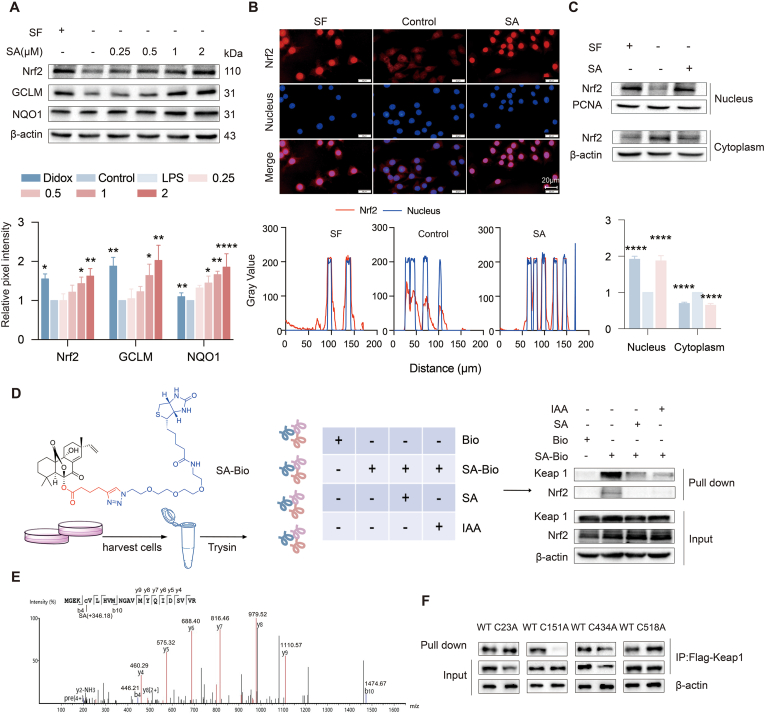
SA activated Nrf2-mediated defensive response in a Keap1-Cys151-dependent manner. (A) SA upregulated Nrf2 and its downstream genes. The protein extraction was analyzed by western blot. (B-C) Cells were treated with 2 μM SA or 4 μM SF for 8 h, followed by nuclear and cytoplasmic protein extraction. Protein level detected by western blot and indirect fluorescence staining. Results are expressed as mean ± SD (n = 3), ∗ indicates significant difference (∗*p* < 0.05, ∗∗*p* < 0.01, ∗∗∗*p* < 0.001). (D) Chemical structure of SA-Bio and pull down Keap1 protein in Beas-2B cells. (E) Mass spectrum of SA binding C151 cysteine in Keap1. (F) The Keap1 with Flag wild-type Keap1 (WT) or Flag mutated Keap1 (C23A, C151A, C434A, and C518A). Protein level detected by western blot.

SA increased the protein levels of Nrf2 and its downstream genes GCLM and NQO1 (Fig. 3A). Translocation of Nrf2 protein from the cytoplasm to the nucleus is an essential step for activation of Nrf2-mediated defense responses. Treatment of SA upregulated the protein level of Nrf2 in the nucleus, which was confirmed by SA-induced Nrf2 nuclear accumulation in immunofluorescence assay (Fig. 3B and C). In addition, SA upregulated the mRNA levels of NQO1, HO-1and GCLM (Fig. S2). To explore and identify the cellular targets of SA, an alkyne-tagged SA-C3 probe was designed and synthesized. The alkyne tag can be further appended with an azide-biotin tag through click chemistry, allowing the cellular targets of SA to be affinity purified for immunoblotting identification. The results showed that Keap1 could be obviously enriched by SA-Bio probe, and was obviously competed out by SA or IAA (Fig. 3D). Therefore, it was proved that SA might bind to cysteine on Keap1 and be further enriched. After incubating the heterologous expression Keap1 protein with SA, LC-MS/MS analysis revealed a SA (+346.18 Da) modification on the cysteine residue at position 151 of Keap1 (Fig. 3E). Keap1 with Flag wild-type Keap1 (WT) or Flag mutant Keap1 (C23A, C151A, C434A and C518A) were designed and introduced into cells for expression. The pull-down experiment was conducted with the SA-Bio probe, and the results demonstrated that SA bound to cysteine 151 of Keap1 (Fig. 3F). Collectively, these data suggested that SA activates Nrf2 by specifically reacting with Cys151 in Keap1.

### 3.4 SA inhibited NF-κB-mediated inflammatory response *in vitro*

**Fig. 4:**
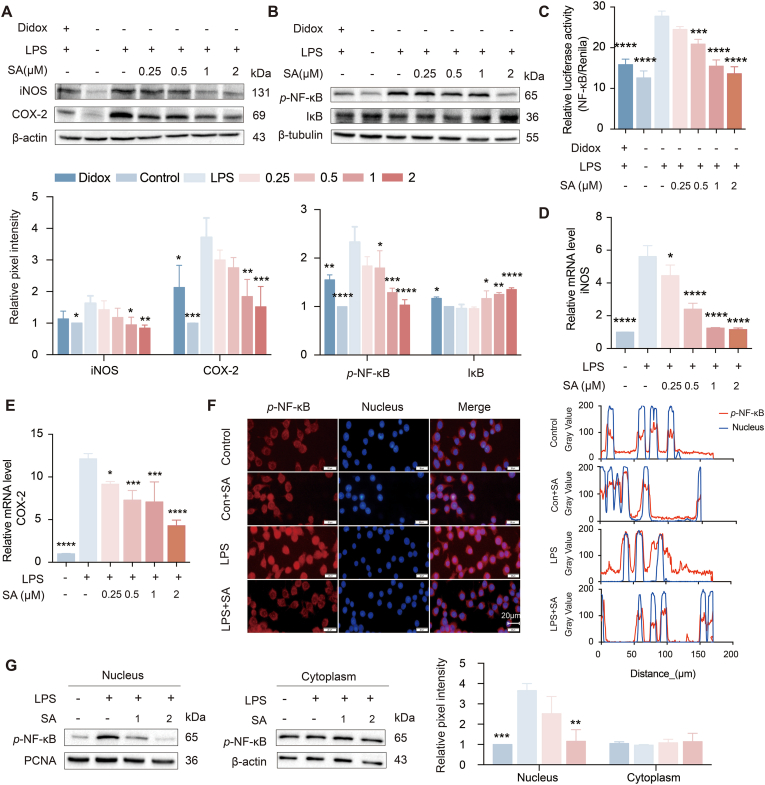
SA inhibited LPS-stimulated activation of NF-κB regulated inflammatory response. (A) The protein levels of iNOS and COX-2 were measured by western blot. Didox (100 μM) was used as a positive control. (B) The protein levels of *p*-NF-κB and IκB were measured by western blot. Didox (100 μM) was used as a positive control. (C) The NF-κB and Renilla luciferase activities were measured. (D-E) The mRNA contents of iNOS and COX-2 were examined with RT-PCR. (F) The indirect fluorescence staining was used to reflect SA inhibited the LPS-induced *p*-NF-κB nuclear translocation. (G) The western blot was used to reflect SA inhibited the LPS-induced *p*-NF-κB nuclear translocation. Results are expressed as mean ± SD (n = 3), ∗ indicates significant difference (∗*p* < 0.05, ∗∗*p* < 0.01, ∗∗∗*p* < 0.001).

The inhibition of SA against NF-κB-regulated-inflammation was investigated using LPS-stimulated RAW 264.7 macrophages. SA was untoxic against RAW264.7 below 5 μM (Fig. S3). At the nontoxic doses, SA dose-dependently inhibited the generation of NO and was more potent than the positive control at 5 μM (Fig. S4). Similarly, treatment with SA (2 μM) significantly blocked the LPS-stimulated increase of *p*-NF-κB, iNOS and COX-2, and reversed the decrease of IκB protein (Fig. 4A and B), and dose-dependently inhibited LPS-stimulated increase of NF-κB luciferase activity (Fig. 4C). Furthermore, LPS-stimulated increases of mRNA levels of iNOS and COX-2 have been dose-dependently reverted after SA treatment (Fig. 4D and E). Next, we evaluated the effect of SA on NF-κB translocation to the nucleus, a critical step for NF-κB activation. As depicted in Fig. 4F and G, treatment with SA (2 μM) significantly inhibited LPS-stimulated nuclear translocation of NF-κB. Thus, these observations suggested that SA inhibited the activation of NF-κB and its downstream genes.

### 3.5 SA inhibited the activation of NLRP3 inflammasome through blocking ASC oligomerization

**Fig. 5:**
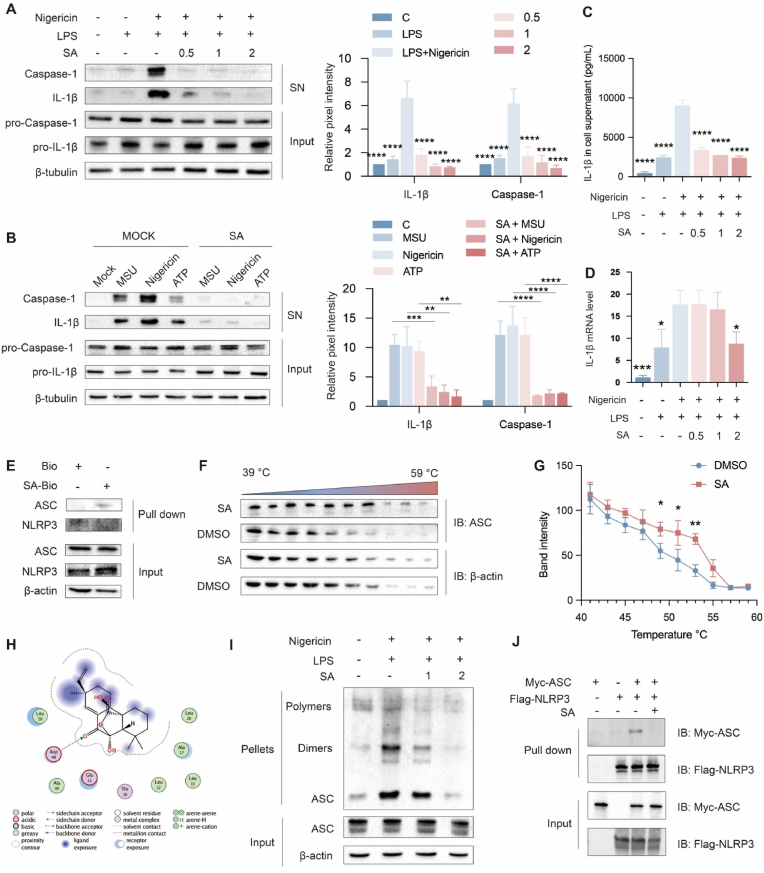
SA inhibited the activation of NLRP3 inflammasome. (A-B) The protein levels of IL-1β and cleaved-caspase-1 after NLRP3 inflammasome induced by different stimulators. The culture supernatant and the total cell lysates were detected by western blot. (C) The levels of IL-1β after NLRP3 inflammasome induced by different stimulators were detected with Elisa kit. (D) The mRNA levels of IL-1β. The mRNA contents were examined with RT-PCR. (E) The SA-Bio probe pull down to enrich ASC or NLRP3 protein. (F) ASC protein stability was measured by CETSA in the cell lysate exposed to SA. (G) The thermal stability quantitative curve of the stability of ASC protein by CETSA assay in cell lysate exposed to SA. Results are expressed as mean ± SD (n = 3), ∗ indicates significant difference (∗*p* < 0.05, ∗∗*p* < 0.01, ∗∗∗*p* < 0.001). (H) Proposed docking modes of SA in the binding site of ASC. (I) Western blot of ASC in the J774A.1 cell with nigericin in the presence or absence of SA. Subsequent testing was performed using ASC oligomerization assay. (J) HEK293T cells were transfected with FLAG-tagged NLRP3 and Myc-tagged ASC in the presence or absence of SA.

J774 A.1 cells were adopted for testing the capability of SA on regulation of NLRP3, and SA under 2 μM had no toxic effect on J774 A.1 cells (Fig. S5). NLRP3 directly regulates the production of downstream IL-1β, IL-18, and caspase-1, and thus is involved in regulating inflammatory process SA at doses of 0.5–2 μM inhibited the increase of protein levels of IL-1β and caspase-1 stimulated by LPS and nigericin. Similarly, SA also blocked the production of caspase-1 and IL-1β stimulated by other NLRP3 stimulators [e.g. monosodium urate crystals (MSU) and ATP], suggesting SA as a broad-spectrum inhibitor of NLRP3 inflammasome (Fig. 5A and B). IL-1β and IL-18, as the secreted protein, can be secreted into cell supernatants and detected by ELISA, the results showed that SA inhibited the overproduction of IL-1β and IL-18 stimulated by LPS together with nigericin, MSU and ATP (Fig. 5C and Fig. S6). In addition, SA decreased mRNA levels of IL-1β and caspase-1(Fig. 5D and Fig. S7). Collectively, SA was an inhibitor of NLRP3 inflammasome in J774A.1 cells.

In order to demonstrate how SA inhibited the NLRP3 inflammasome, the pathway proteins were enriched with SA-Bio probe, and the results showed that SA directly binds to ASC (Fig. 5E). Cellular thermal shift assay has been validated that SA efficiently protected ASC against temperature-dependent degradation and enhanced the thermal stability of ASC (Fig. 5F and G). In addition, the molecular docking results showed that SA formed hydrogen bonds with ASP48 of the ASC protein with binding energies −6.3 kcal mol^−1^, and hydrophobic interactions existed between SA and LEU15, LEU20, ALA49, and LEU50 residues, which allowed SA to bind stably to the pocket of ASC (Fig. 5H and Fig. S8). Further research found that following LPS-nigericin stimulation, NLRP3 could interact with ASC, and their protein interaction was significantly impaired after the SA treatment. Moreover, SA could suppress nigericin-induced ASC oligomerization, leading to further inhibition of NLRP3 activation (Fig. 5I and J). These data suggested that SA blocked ASC oligomerization to suppress NLRP3 activation.

### 3.6 SA inhibited activations of NLRP3 and NF-κB in an Nrf2-dependent manner

**Fig. 6:**
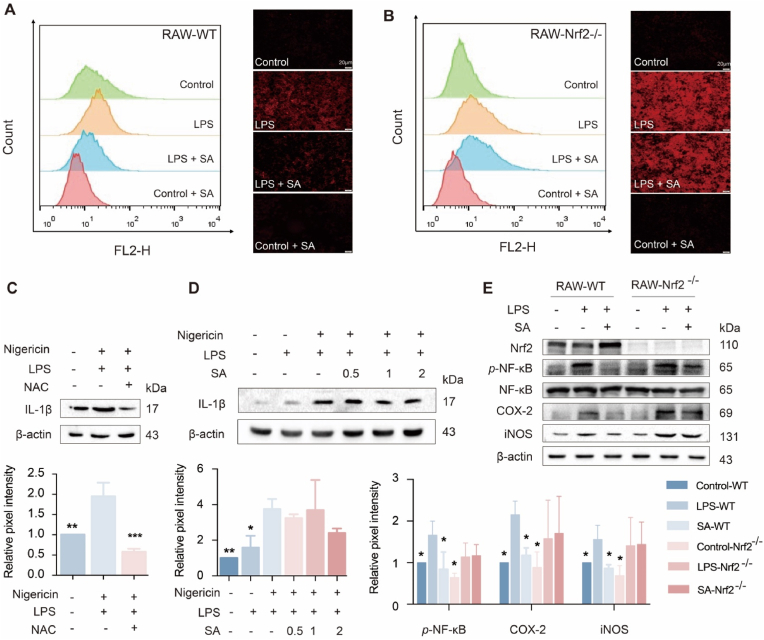
Mechanism of action of SA. (A) ROS level in RAW 264.7 cells. (B) ROS level in Nrf2^−/−^ RAW 264.7 cells. (C) Effect of NAC on the protein expressions of IL-1β in J774A.1 cells. (D) Effect of SA on the protein expressions of IL-1β in J774A.1 cells transfected with siRNA. (E) Effect of SA on the protein expressions of *p*-NF-κB in Nrf2^−/−^ RAW 264.7 cells or WT RAW 264.7 cells. Results are expressed as mean ± SD (n = 3), ∗ indicates significant difference (∗*p* < 0.05, ∗∗*p* < 0.01, ∗∗∗*p* < 0.001).

Excessive production of ROS exists in pneumonia, amplifies inflammatory response and leads to the deterioration of the disease. SA (2 μM) significantly attenuated the LPS-induced overproduction of ROS in RAW 264.7 cells (Fig. 6A). However, when cells transfected with siRNA-Nrf2 to suppress endogenous Nrf2 expression, the ability of SA to inhibit the LPS-induced upregulation of ROS disappeared (Fig. 6B), suggesting that SA inhibited ROS generation by activating Nrf2-mediated antioxidant system. We have found that the ROS scavenger N-acetylcysteine (NAC) inhibited IL-1β secretion [25], indicating that ROS is involved in the NLRP3 activation (Fig. 6C). Furthermore, SA failed to reduce protein levels of IL-1β in cells transfected with Nrf2-siRNA (Fig. 6D), implying that inhibition of NLRP3 by SA depended on Nrf2-regulated ROS production. Next, we examined the role of Nrf2 in SA-induced inhibition of NF-kB using Nrf2^−/−^ RAW 264.7 cells. As depicted in Fig. 6E, SA reverted LPS-stimulated upregulation of the protein levels of *p*-NF-κB, COX-2 and iNOS in WT-RAW 264.7 cells, whereas this effect has vanished in Nrf2^−/−^ cells. Therefore, SA inhibited NLRP3 and NF-κB in an Nrf2-dependent manner.

### 3.7 SA attenuated pneumonia predominantly in an Nrf2-dependent manner

**Fig. 7:**
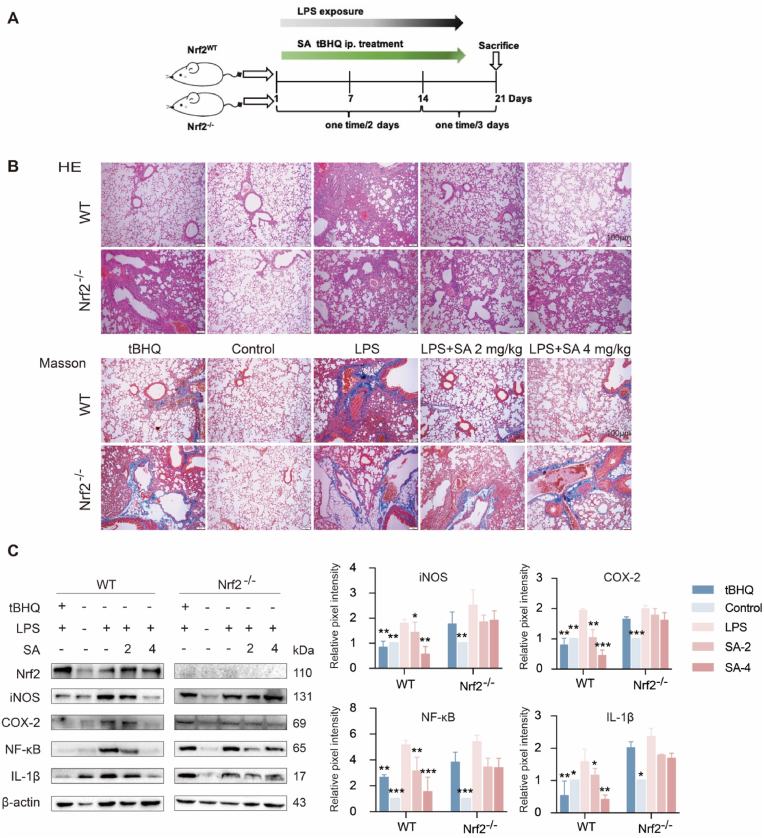
SA attenuated pneumonia predominantly in Nrf2-WT but not Nrf2^−/−^ mice. (A) Animal test protocol. tBHQ (40 mg/kg), and SA (2 and 4 mg/kg) were injected intraperitoneally for 4 h followed by stimulation with LPS (10 mg/kg) once every two days for 2 weeks, once every 3 days for 1 week at the third week. The mice were sacrificed 24 h after LPS nebulization. (B) Lung tissue sections were stained with H&E or Masson. (C) Western blot was performed to detect the expression of inflammatory pathway proteins in lung tissues. Results are expressed as mean ± SD (n = 3), ∗ indicates significant difference (∗*p* < 0.05, ∗∗*p* < 0.01, ∗∗∗*p* < 0.001).

To evaluate whether Nrf2 is involved in SA reduction of acute pneumonia in mice, Nrf2^−/−^ mice were employed. After three weeks of dosing, the lung tissues were collected for the subsequent analysis (Fig. 7A). Through HE staining and Masson's staining, SA showed an obvious anti-inflammatory effect by reversal of significant lung inflammation by LPS, including thickening and collapse of alveolar walls, and inflammatory cell infiltration in Nrf2-WT mice, but not in Nrf2^−/−^ mice. In Nrf2-WT mice, the anti-inflammatory effect of SA (4 mg/kg) was similar to that of the positive control tBHQ (Fig. 7B). Furthermore, SA administration induced a notable decline of iNOS, COX-2 and NF-κB expression in Nrf2-WT mice compared with Nrf2^−/−^ mice. SA-mediated decrease in proinflammatory cytokines IL-1β in Nrf2-WT mice was hugely antagonized in Nrf2^−/−^ mice (Fig. 7C). Collectively, these results indicated that SA attenuated pneumonia predominantly in an Nrf2-dependent manner.

## 4 Discussion

Oxidative stress and inflammatory response, play a crucial role in the onset and progression of pneumonia, one of the most common lung diseases [26,27]. Usually, Keap1-Nrf2 axis are abnormally activated under oxidative stress. In response to strss, sensor cysteines within Keap1 allows Nrf2 to escape ubiquitination and be translocated to the nucleus to initiate the antioxidant transcription program [28,29]. In our study, we found a novel Nrf2 agonist SA, from *Sphaeropsis sapinea* f. sp. *cupressi*, had the potential to treat oxidative stress-related diseases [22]. In our study, SA showed activity in a mouse model of acute pneumonia induced by aerosol administration of LPS. Notably, the effective dose of SA is only 2 mg/kg, which is comparable to tBHQ (40 mg/kg). To gain insight into the key pathways, the results of transcriptome sequencing analysis indicated that SA could be associated with regulations of Nrf2, NF-κB and NLRP3 inflammasome. Therefore, SA is a potent inhibitor of LPS-induced acute pneumonia in mice, making it a promising candidate for the treatment of pneumonia-related diseases.

Nrf2 is a critical transcription factor, and Nrf2 activation could be a strategy for treating respiratory diseases, like asthma and ALI [30]. Activation of Nrf2 could regulate the synthesis of endogenous antioxidant enzymes (e.g. NQO1, GCLM) and inflammation-related factors (e.g. IL-1β) [31]. In the previous research, we indicated that SA could induce antioxidant proteins in Beas-2B cells and inhibit the overproduction of ROS [22]. In the present study, we confirmed that SA could significantly reduce the production of ROS, promote Nrf2 translocation into the nucleus, and up-regulate the expression of downstream proteins, e.g. GCLM, NQO1 *in vivo* and *in vitro*. Besides, based on the role of NF-κB in the expression of proinflammatory genes including cytokines, chemokines, and adhesion molecules, NF-κB was considered as a major regulator of inflammation. Our results also showed that SA was able to inhibit NF-κB gene transcription, and block the activation of NF-κB and its downstream genes, e.g. iNOS and COX-2 *in vitro* and *in vivo*. Beyond that, NLRP3 inflammasome regulates the occurrence of pyroptosis, and promotes the release of inflammatory factors, which in turn triggers oxidative stress in the lungs [32,33]. We found that SA could significantly inhibit the NLRP3 inflammasome of macrophages, reduce the release of IL-1β. Above all, SA attenuated acute pneumonia by regulating of Nrf2, NF-κB and NLRP3 inflammasome.

**Fig. 8:**
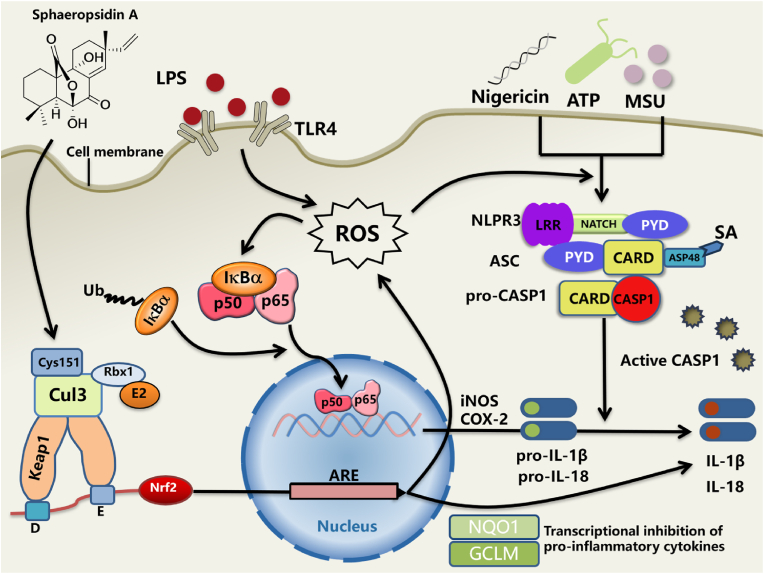
Connection of Nrf2, NF-κB, NLRP3 Pathways and the mechanism of SA. When NLRP3 agonists or ROS stimulate cells, they activate the adaptor protein ASC, and its PYD domain recruits NLRP3. The CARD domain of NLRP3 binds to the CARD domain of Pro-caspase-1 to self-cleave and activate Pro-caspase-1 and generate a series of inflammatory factors. When cells are subjected to external stimuli such as LPS, LPS binds to TLR4 receptors on the cell surface and generates endogenous ROS, prompting NF-κB translocation into the nucleus to exert inflammation and inducing the generation of downstream inflammatory proteins and pro-inflammatory factors. Under normal conditions, Keap1 and Nrf2 bind together, and when Nrf2 activator or endogenous ROS stimulate cells, they lead to the release of Nrf2 from Keap1, translocating Nrf2 into the nucleus and activating antioxidant ARE sequences, thereby inducing a series of downstream antioxidant enzymes.

Given the strong link among ROS, NF-κB pathway, and NLRP3 inflammasome, the results that SA could inhibit the generation of excessive ROS explained the interaction between three pathways mentioned above. Increasing evidence showed that excessive ROS activates NF-κB and NLRP3, while accumulation of ROS contributes to phosphorylation and degradation of IKBα, releasing and translocating of NF-κB to the nucleus [34,35]. Moreover, activation of the Nrf2 pathway regulates antioxidant protein expression to scavenge ROS and effectively block oxidative stress [36]. In our study, SA effectively reduces the level of ROS, thereby inhibiting the activation of NF-κB and NLRP3 inflammasome. When Nrf2 is silenced in macrophage, the abilities of SA to clear excessive ROS and inhibit the activation of NF-κB and NLRP3 inflammasome disappeared. Furthermore, by the konckout of Nrf2 in mice, the inhibition of SA in NLRP3 inflammasome and NF-κB activation also disappeared. In all, these data indicate that SA inhibited the NLRP3 inflammasome and NF-κB pathway by reducing the excess production of ROS through Nrf2 activation (Fig. 8).

Keap1 is a partner of Nrf2 and Keap1 regulates Nrf2 ubiquitination and transport to the nucleus [17,37]. In our previous studies, SA was identified as an Nrf2 agonist, however, the activation mechanism hasn't been studied thoroughly. Here, we found that SA activates the Nrf2 signaling pathway by covalently modifying Keap1 on cysteine residues. The pull-down experiment showed that SA directly bound to Keap1. Usually, Nrf2 is activated through active sites covalently bound to KEAP1-specific cysteine by alpha, beta unsaturated ketones, such as Cys151, Cys273, and Cys288 [17,38]. Cysteine electrophilic Keap1-Nrf2 inhibitor that covalently binds to Keap1 can effectively enhance the antioxidant defense response, for example, dimethyl fumarate (Tecfidera) is clinically used to treat multiple sclerosis and psoriasis [39,40]. Meanwhile, we found that SA could bind to Cys151 using LC-MS/MS and cysteine mutation techniques. Cys151 is located in the BTB domain and affects the interaction of Cul3 and Keap1 [40,41]. Thus, SA can modify Keap1 by forming covalent bonds with Cys151. SA interacted with Cys151, causing Cul3 to separate from the Keap1-BTB domain, facilitating Nrf2 translocation into the nucleus. In addition, NLRP3 recruits ASC and binds to pro-caspase-1, which is essential for the activation of caspase-1 and cleavage of pro-IL-1β [42]. Here, our data showed that SA inhibited NLRP3 inflammasome activation and down-regulate its downstream mediators (e.g. IL-1β, Caspase-1). SA formed intermolecular hydrogen bonds with ASP48 of ASC, inhibited the oligomerization of ASC, and then inhibited the activation of NLRP3 validation bodies. Hence, SA can not only form covalent bonds with Keap1, but also form intermolecular hydrogen bonds with ASC. These two interactions explained the mechanism how SA treat LPS-induced acute pneumonia in mice.

## 5 Conclusions

Collectively, our findings reveal that SA is a novel molecule covalently binding to Keap1 that activates Nrf2. It is also a potent inhibitor in LPS-induced acute pneumonia mice. This makes it possible to develop lead compounds for covalently bound to Keap1 as well as therapeutic candidates for pneumonia-related diseases.

## CRediT authorship contribution statement

**Kang Yang:** Data curation, Formal analysis, Methodology. **Qing-Tong Han:** Data curation, Formal analysis, Methodology, Project administration, Writing – original draft. **Rong-Xue Xing:** Formal analysis, Methodology. **Zhi-Ying Li:** Data curation, Formal analysis, Methodology. **Lin-Tao Xu:** Formal analysis, Methodology. **Lu-Zhou Chen:** Formal analysis, Software. **Lan Xiang:** Resources, Writing – review & editing. **Dong-Mei Ren:** Supervision, Validation, Writing – review & editing. **Qing-Wen Hu:** Writing – review & editing. **Xiao-Ning Wang:** Project administration, Writing – review & editing. **Tao Shen:** Funding acquisition, Writing – review & editing, Conceptualization, Project administration, Resources, Validation.

## Declaration of competing interest

The authors declare that there are no competing financial interests.
