# Supplementary material for: Sphaeropsidin A covalently binds to Cys 151 of Keap1 to attenuate LPS-induced acute pneumonia in mice
Source: Redox Biol. 2025 Mar 27;82:103621. doi: 10.1016/j.redox.2025.103621 (PMC11999469; doi:10.1016/j.redox.2025.103621)
Supplement: Multimedia component 1 [file mmc1.docx]

**Supporting Information for**

**Sphaeropsidin A covalently binds to Cys 151 of Keap1 to attenuate LPS-induced acute pneumonia in mice**

Kang Yang ^a,b,c,d,1^, Qing-Tong Han ^a,b,c,d,1^, Rong-Xue Xing ^a,b,c,d^, Zhi-Ying Li ^a,b,c,d^, Lin-Tao Xu ^a,b,c,d^, Lu-Zhou Chen ^a,b,c,d^, Lan Xiang ^a,b,c,d^, Dong-Mei Ren ^a,b,c,d^, Qing-Wen Hu ^d^, Xiao-Ning Wang ^a,b,c,d,**^, Tao Shen ^a,b,c,d,*^

^a^ *State Key Laboratory of Discovery and Utilization of Functional Components in Traditional Chinese Medicine, Shandong University, Jinan 250012, China*

^b^ *Key Lab of Chemical Biology (MOE), School of Pharmaceutical Sciences, Shandong University, Jinan 250012, China*

^c^ *Shandong Engineering Research Center for Traditional Chinese Medicine Standard, School of Pharmaceutical Sciences, Shandong University, Jinan 250012, China*

^d^ *Shandong Key Laboratory of Bioactive Components and Translational Research of Traditional Chinese Medicine, Jinan 250012, China*

^*^ Corresponding author, School of Pharmaceutical Sciences, Shandong University, No. 44, Wenhua Xi Road, Jinan City, Shandong Province, 250012, China.

^**^ Corresponding author, School of Pharmaceutical Sciences, Shandong University, No. 44, Wenhua Xi Road, Jinan City, Shandong Province, 250012, China.

*E-mail addresses:* [shentao@sdu.edu.cn](mailto:shentao@sdu.edu.cn) (Tao Shen), [wangxn@sdu.edu.cn](mailto:wangxn@sdu.edu.cn) (Xiao-Ning Wang)

^1^ These authors made equal contributions to this work.

**Chemical synthesis**

**Synthesis of SA-C3**

Under nitrogen, a solution of 5-hexynoic acid (33 mg, 0.29 mmol), EDCI (84 mg, 0.44 mmol) and DMAP (14 mg, 0.12 mmol) in 5 mL DCM was stirred at room temperature for 2 hours. Then, SA (20 mg, 0.06 mmol) in 2 mL DCM was added and stirred for another 4 hours. The mixture was suspended in DCM and water, and the DCM phase was collected. Then extracted with another 10 mL DCM for twice. Combined the organic phase and dried with anhydrous Na_2_SO_4_. The desired compound was purified by flash chromatography using PE/EtOAc (5:1) to obtin SA-C3 (14.8 mg, 56.0%) as a white solid .^1^H NMR (400 MHz, CDCl_3_) *δ*: 6.54 (d, *J* = 1.7 Hz, 1H), 5.83 (dd, *J* = 17.7, 10.6 Hz, 1H), 5.08-5.04 (m, 2H), 2.86 (s, 1H), 2.68 (m, 2H), 2.35 (dt, *J* = 6.9, 2.6 Hz, 2H), 1.99 (t, *J* = 2.64 Hz, 1H), 1.97-1.25(m, 12H), 1.20 (s, 3H,), 1.11 (s, 3H), 1.08 (s, 3H); ^13^C NMR (100 MHz, CDCl_3_) *δ*: 189.61, 174.11, 169.36, 148.49, 144.80, 135.94, 113.09, 105.45, 83.17, 71.70, 69.50, 56.21, 50.97, 40.63, 38.93, 33.08, 32.83, 32.71, 29.70, 26.72, 24.50, 23.48, 22.72, 22.47, 17.88, 17.77. HR-MS: C_26_H_32_NaO_6_ for [M+Na]^+^, calculated 463.2097; found, 463.2096.

**Synthesis of SA-Bio**

Under nitrogen, a solution of SA-C3 (8 mg, 0.018 mmol), Biotin-PEG-N_3_ (8 mg, 0.018 mmol) and CuSO_4_ (2 mg, Cat) was stirred in 2 mL DMSO, sodium ascorbate (35 mg, 0.18 mmol) was added and the mixture was stirred until the starting materrials were consumed. The mixture was extracted with DCM and purified by P-TLC to obitin SA-Bio (7.5 mg, 47.1%) as a white solid. ^1^H NMR (400 MHz, CDCl_3_) *δ*: 7.68 (s, 1H), 6.86 (t, *J* = 5.3 Hz, 1H), 6.35 (s, 1H), 5.83-5.76 (m, 2H), 5.28 (s, 1H), 5.08-4.99 (m, 2H), 4.53 (t, *J* = 4.6 Hz, 2H), 4.50-4.47 (m, 1H), 4.36-4.33 (m, 1H), 3.89-3.85 (m, 2H), 3.65-3.52 (m, 10H), 3.44-3.37 (m, 2H), 3.17-3.12 (m, 1H), 2.86 (s, 1H), 2.89-2.86 (m, 2H), 2.68 (m, 2H), 2.10-2.07 (m, 2H), 1.97-1.25(m, 18H), 1.20 (s, 3H,), 1.11 (s, 3H), 1.08 (s, 3H); ^13^C NMR (100 MHz, CDCl_3_) *δ:* 183.86, 174.57, 173.48, 169.46, 164.07,147.79, 144.80, 131.95, 123.15, 113.88, 106.54, 71.36, 70.64, 70.48, 70.20, 69.93, 69.58, 61.83, 60.18, 56.60, 55.52, 53.39, 50.28, 40.66, 40.33, 39.29, 38.95, 36.02, 33.69, 32.75, 29.56, 28.27, 26.88, 25.97, 24.68, 24.45, 23.66, 22.51, 17.92. HR-MS: C_44_H_64_N_6_NaO_11_S for [M+Na]^+^, calculated 907.4251; found, 907.4252.


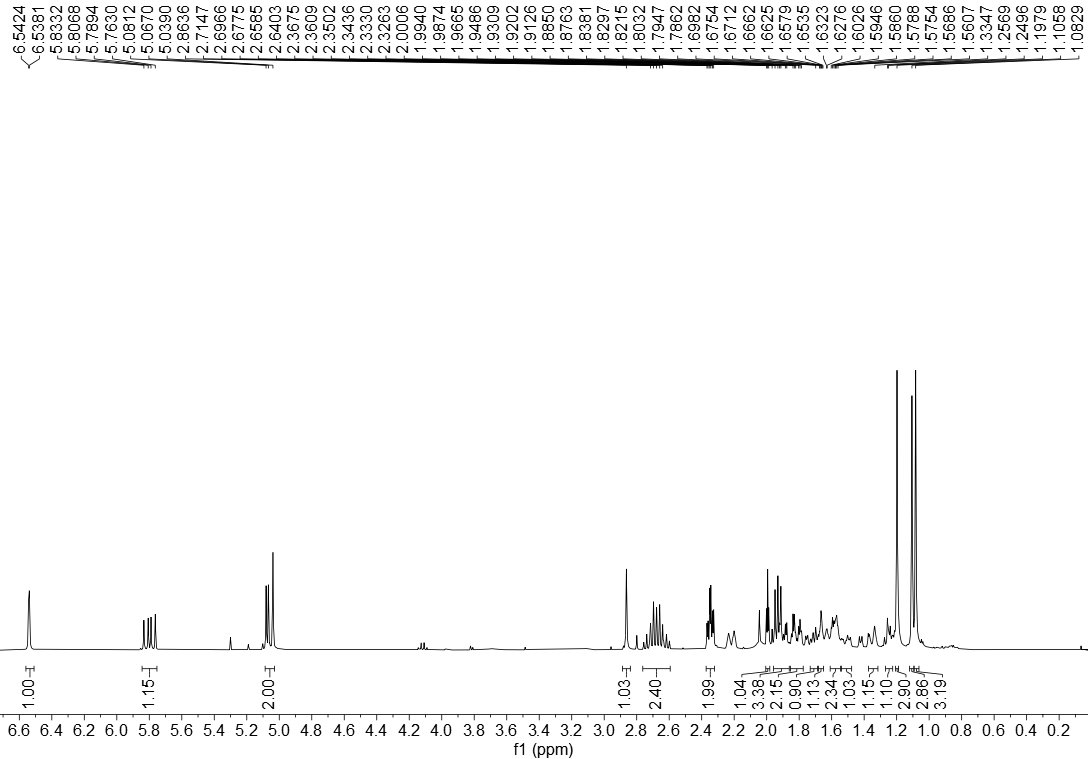


^1^H NMR of **SA-C3** in CDCl_3_


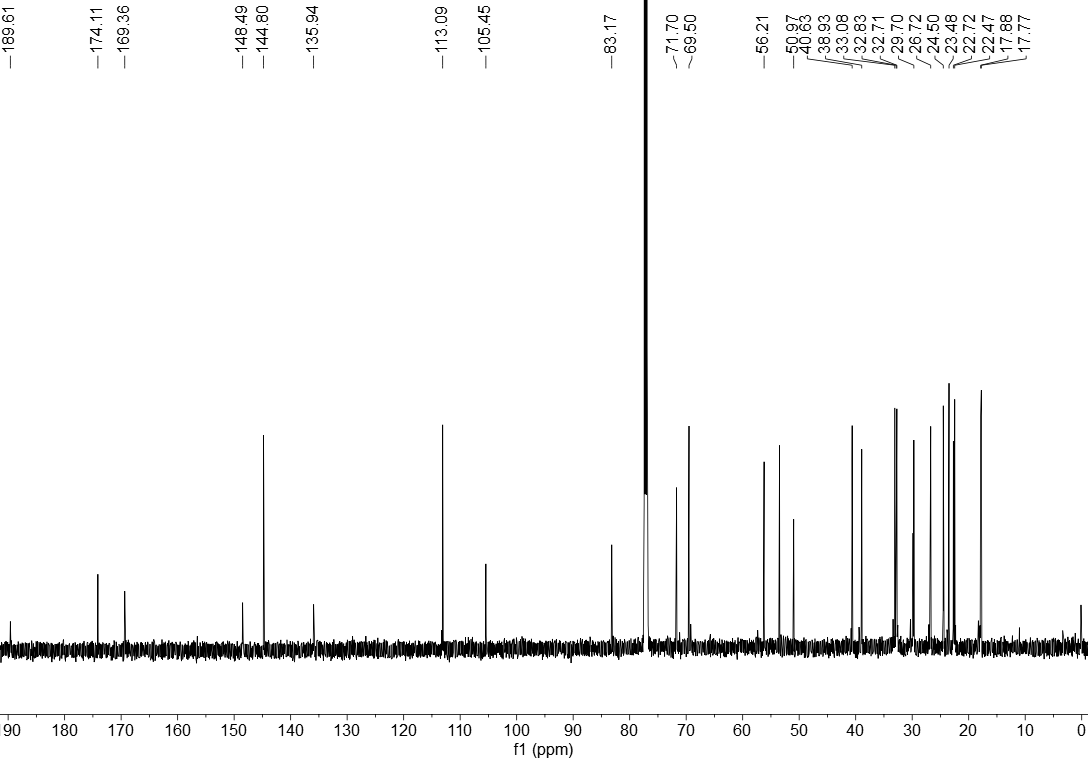


^13^C NMR of **SA-C3** in CDCl_3_


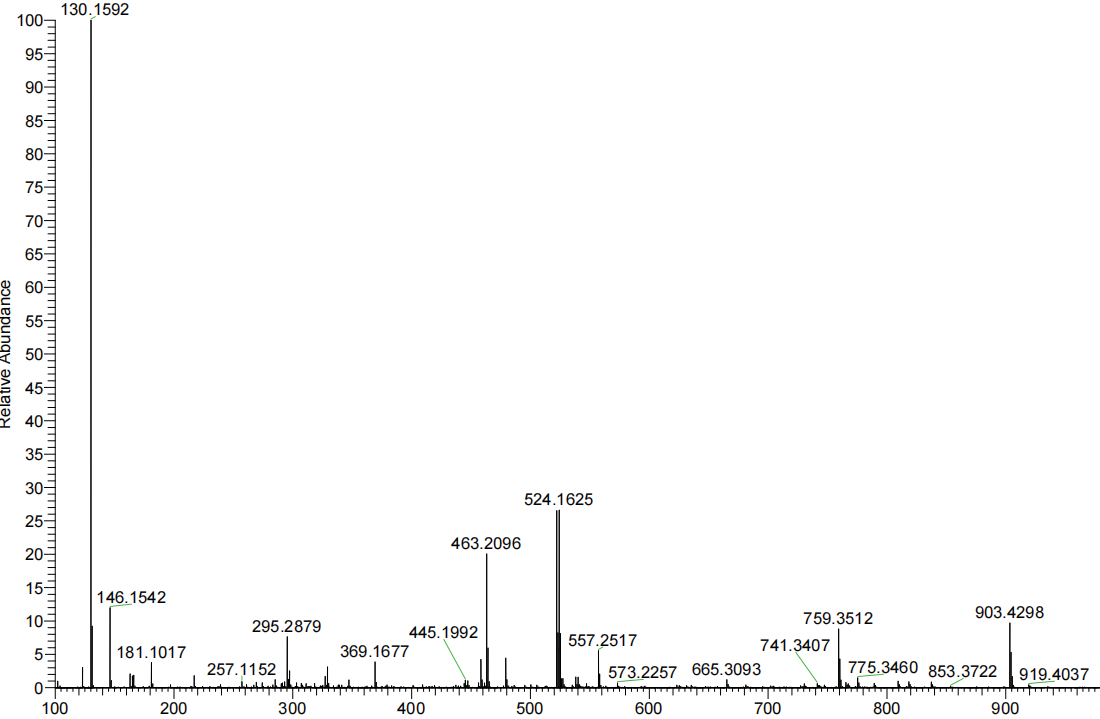


HR MS of **SA-C3**


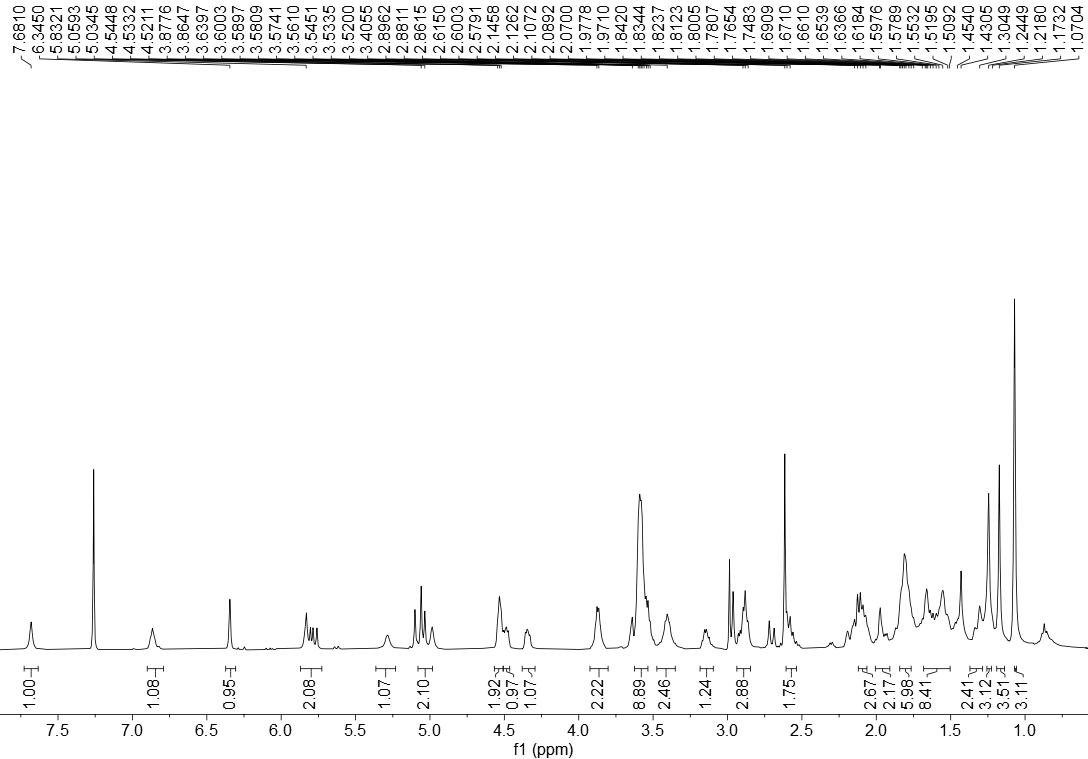
^1^H NMR of **SA-Bio** in CDCl_3_


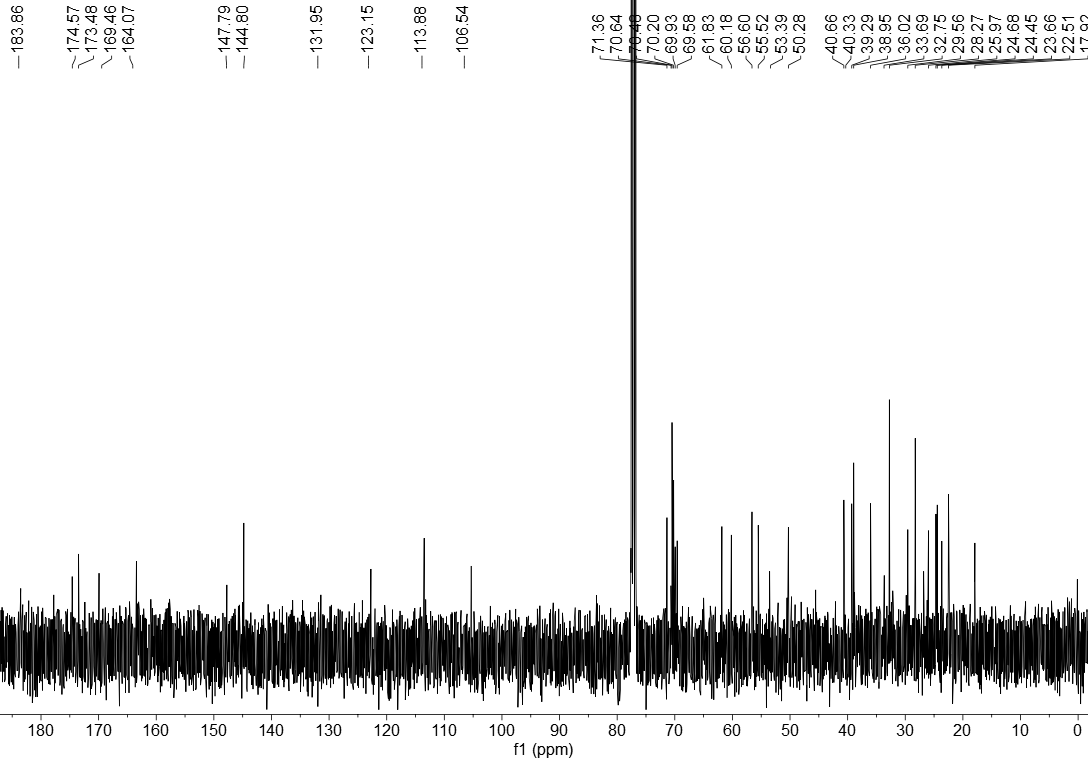


^13^C NMR of **SA-Bio** in CDCl_3_


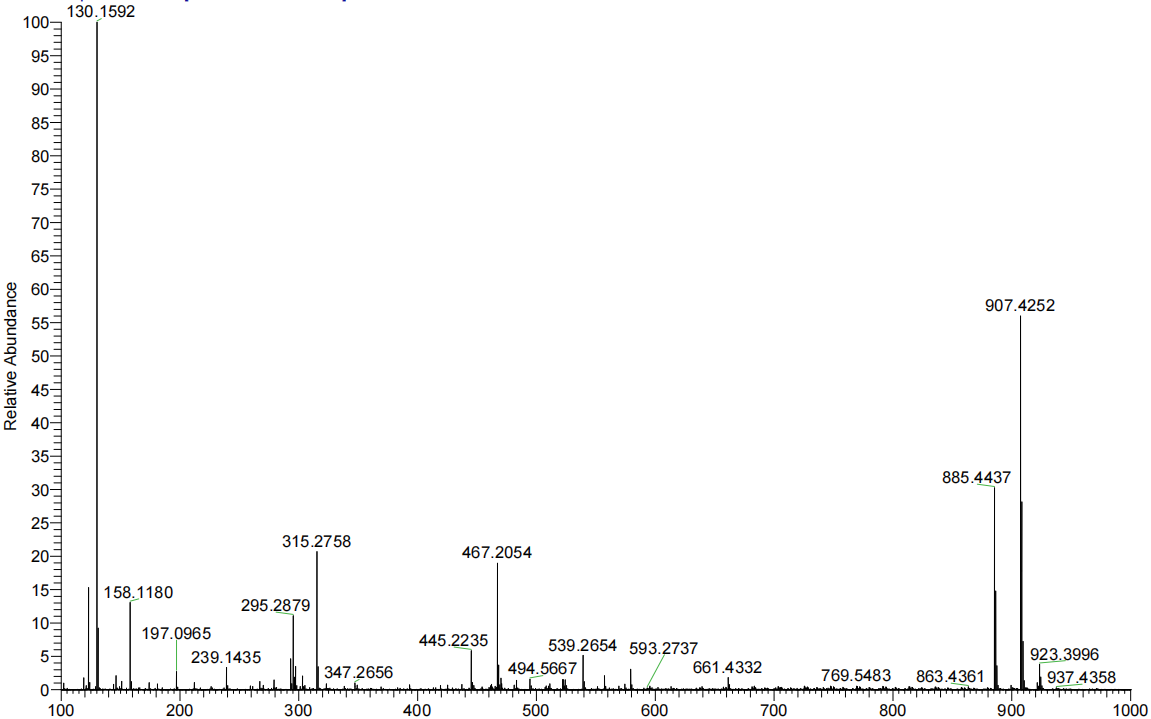


HR MS of **SA-Bio**

**Supplementary Table 1: Primers used for real-time PCR**

| Name | Sequence (5’-3’) |
| --- | --- |
| miNOS, forward | 5′-CAAGAGTTTGACCAGAGGACC-3′ |
| miNOS, reverse | 5′-TGGAACCACTCGTACTTGGGA-3′ |
| mCOX-2, forward | 5′-CACTACATCCTGACCCACTT-3′ |
| mCOX-2, reverse | 5′-ATGCTCCTGCTTGAGTATGT-3′ |
| mβ-actin, forward | 5′-CGCTCATTGCCGATAGTGAT-3′ |
| mβ-actin, reverse | 5′-TGTTTGAGACCTTCAACACC-3′ |
| mCaspase-1, forward | 5′-ACAAGGCACGGGACCTATG-3′ |
| mCaspase-1, reverse | 5′-TCCCAGTCAGTCCTGGAAATG-3′ |
| mIL-1β, forward | 5′-TTACAGTGGCAATGAGGATGAC-3′ |
| mIL-1β, reverse | 5′-GTCGGAGATTCGTAGCTGGAT-3′ |
| m GAPDH, forward | 5′- AAGGTCGGAGTCAACGGATTT -3′ |
| m GAPDH, reverse | 5′- AGATGATGACCCTTTTGGCTC -3′ |
| mGCLM, forward | 5′- TTGGAGTTGCACAGCTGGATT -3′ |
| mGCLM, reverse | 5′- TGGTTTTACCTGTGCCCACTG -3′ |
| mNQO1, forward | 5′- TATCCTTCCGAGTCATCTCTAGCA -3′ |
| mNQO1, reverse | 5′- TCTGCAGCTTCCAGCTTCTTG -3′ |
| mHO-1, forward | 5′- GAGCAGAACCAGCCTGAACTA -3′ |
| mHO-1, reverse | 5′- GGTACAAGGAAGCCATCACCA -3′ |
| Myc-ASC, forward | 5′-GATATAAGCTTACCATGGAGCAGAAACT CATCTCTGAAGAGGATCTGGGGCGCGC GCGCG-3′ |
| Myc-ASC, reverse | 5′- GATATCTCGAGTCAGCTCCGCTC  CAGGTC -3′ |
| FLAG-NLRP3, forward | 5′- ATGAAGATGGCAAGCACCCG -3′ |
| FLAG-NLRP3, reverse | 5′- CGTCATCCTTGTAATCCCAAGAAGGCTCA AAGACGACG -3′ |


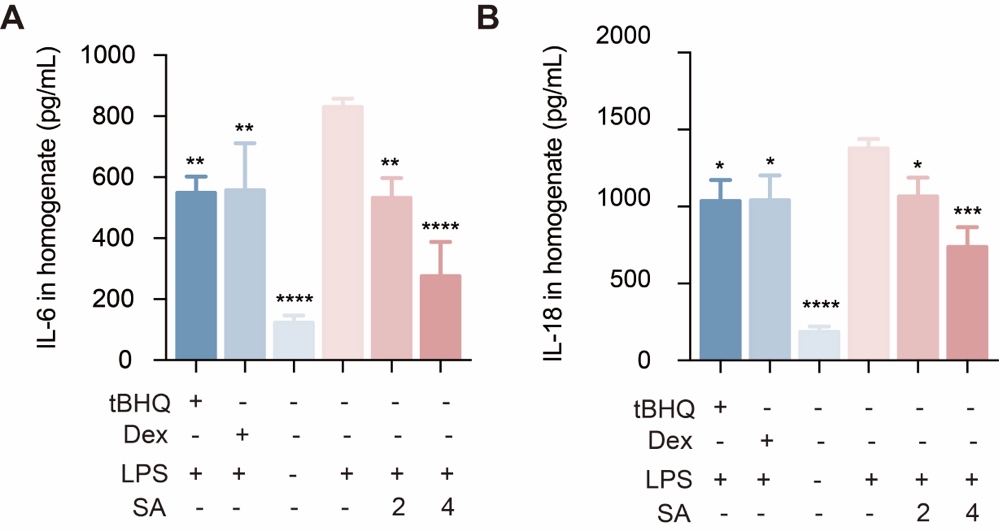


**Supporting Information Fig. S1** (A) The levels of IL-6 in BALF. (B) The levels of IL-18 level in BALF. They were determined using ELISA kit. Results are expressed as mean ± SD (n = 3), * indicates significant difference (**p* < 0.05, ***p* < 0.01, ****p* < 0.001).


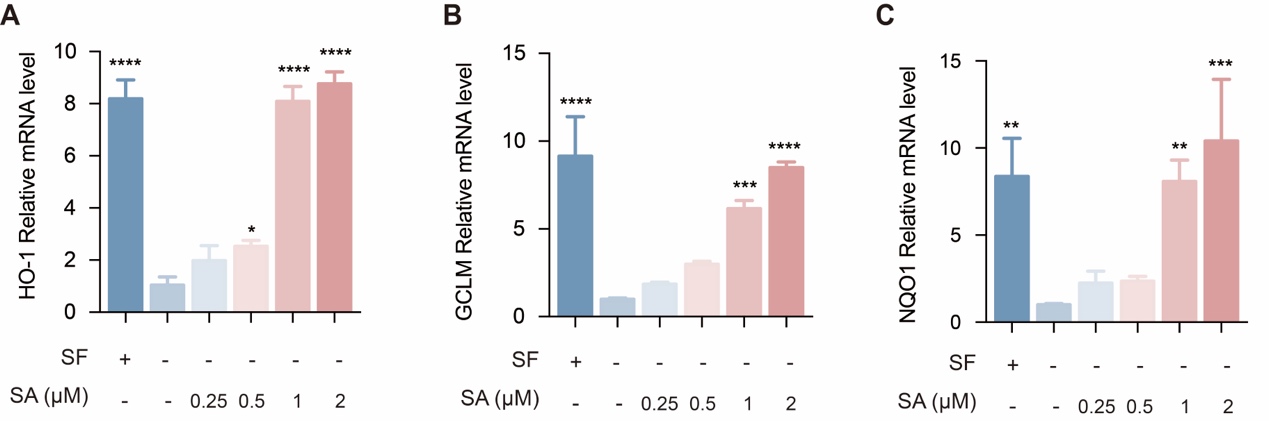


**Supporting Information Fig. S2** The mRNA levels of HO-1 (A), GCLM (B) and NQO1(C). The mRNA contents were examined with RT-PCR. Results are expressed as mean ± SD (n = 3), * indicates significant difference (**p* < 0.05, ***p* < 0.01, ****p* < 0.001).


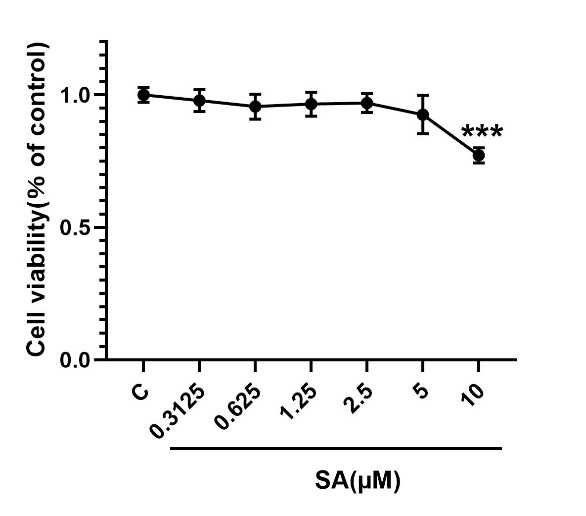


**Supporting Information Fig. S3** Absorbance was measured after RAW 264.7 cells were treated with different concentrations of SA. Results are expressed as mean ± SD (n = 3), * indicates significant difference (****p* < 0.001).


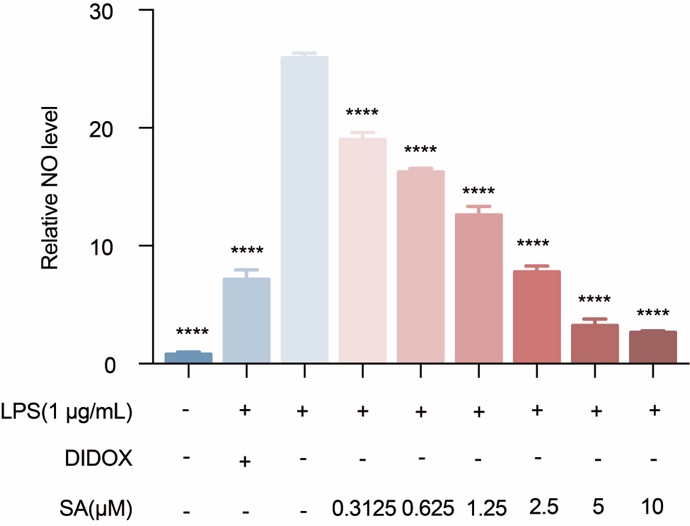


**Supporting Information Fig. S4** Relative NO level in the culture supernatant of RAW 264.7 cells. Results are expressed as mean ± SD (n = 3), * indicates significant difference (**p* < 0.05, ***p* < 0.01, ****p* < 0.001).


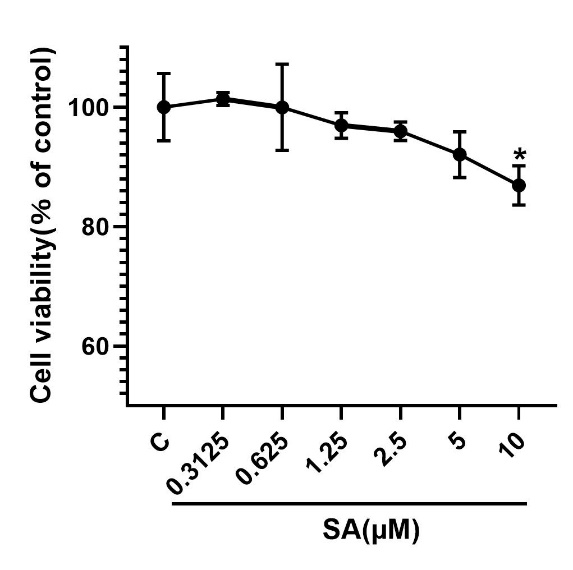


**Supporting Information Fig. S5** Absorbance was measured after J774A.1 cells were treated with different concentrations of SA. Results are expressed as mean ± SD (n = 3), * indicates significant difference (**p* < 0.05).


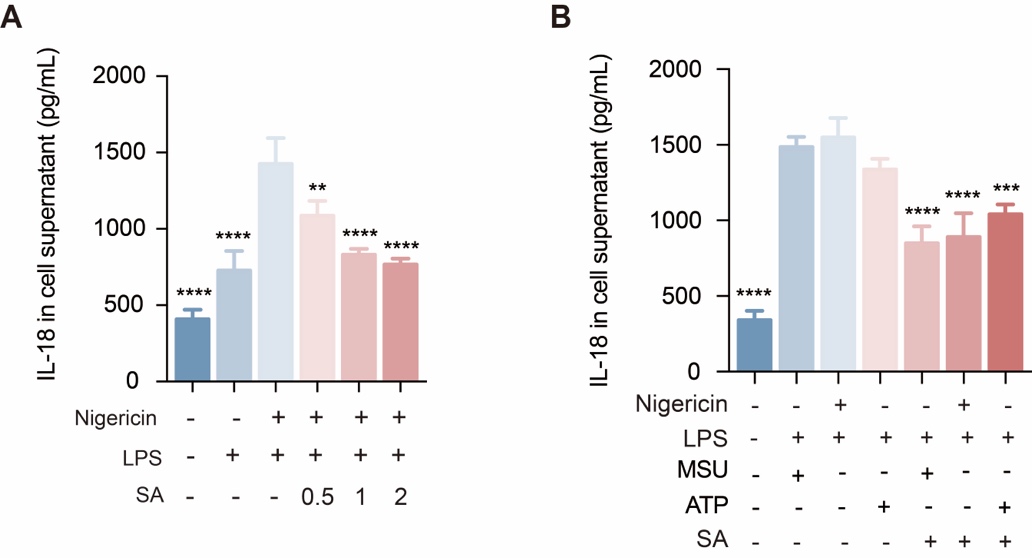


**Supporting Information Fig. S6** The levels of IL-18 (A-B) after NLRP3 inflammasome induced by different stimulators were detected with Elisa kit. Results are expressed as mean ± SD (n = 3), * indicates significant difference (**p* < 0.05, ***p* < 0.01, ****p* < 0.001).

**Supporting Information Fig. S7** The mRNA levels of Caspase-1. The mRNA contents were examined with RT-PCR. Results are expressed as mean ± SD (n = 3), * indicates significant difference (**p* < 0.05, ***p* < 0.01, ****p* < 0.001).


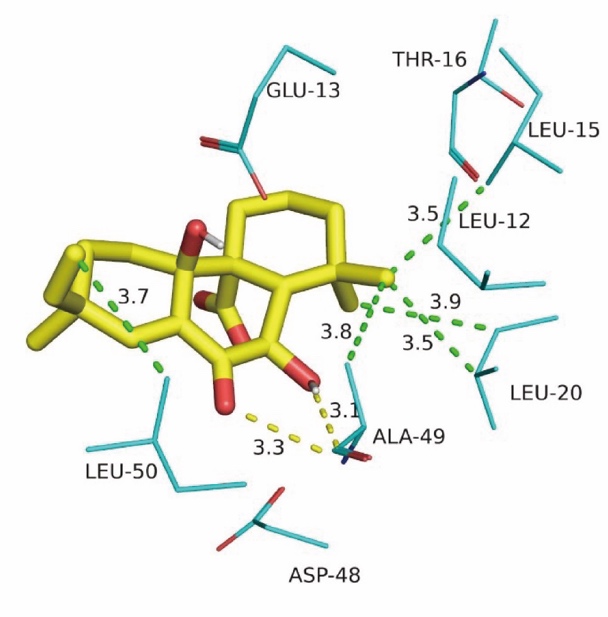


**Supporting Information Fig. S8** Hydrophobic interactions existed between SA and LEU15, LEU20, ALA49, and LEU50 residues.
